# An AVMD-DBN-ELM Model for Bearing Fault Diagnosis

**DOI:** 10.3390/s22239369

**Published:** 2022-12-01

**Authors:** Xue Lei, Ningyun Lu, Chuang Chen, Cunsong Wang

**Affiliations:** 1 College of Automation Engineering, Nanjing University of Aeronautics and Astronautics, Nanjing 211106, China; 2 State Key Laboratory of Mechanics and Control of Mechanical Structures, Nanjing University of Aeronautics and Astronautics, Nanjing 211106, China; 3 College of Electrical Engineering and Control Science, Nanjing Tech University, Nanjing 211816, China; 4 Institute of Intelligent Manufacturing, Nanjing Tech University, Nanjing 210009, China

**Keywords:** bearing fault diagnosis, variable working conditions, adaptive VMD, mode sorting, DBN-ELM

## Abstract

Rotating machinery often works under complex and variable working conditions; the vibration signals that are widely used for the health monitoring of rotating machinery show extremely complicated dynamic frequency characteristics. It is unlikely that a few certain frequency components are used as the representative fault signatures for all working conditions. Aiming at a general solution, this paper proposes an intelligent bearing fault diagnosis method that integrates adaptive variational mode decomposition (AVMD), mode sorting based deep belief network (DBN) and extreme learning machine (ELM). It can adaptively decompose non-stationery vibration signals into temporary frequency components and sort out a set of effective frequency components for online fault diagnosis. For online implementation, a similarity matching method is proposed, which can match the online-obtained frequency-domain fault signatures with the historical fault signatures, and the parameters of AVMD-DBN-ELM model are set to be the same as the most similar case. The proposed method can decompose vibration signals into different modes adaptively and retain effective modes, and it can learn from the idea of an attention mechanism and fuse the results according to the weight of MIV. It also can improve the timeliness of the fault diagnosis. For comprehensive verification of the proposed method, the bearing dataset from the University of Ottawa is used, and some recent methods are repeated for comparative analysis. The results can prove that our proposed method has higher reliability, higher accuracy and higher efficiency.

## 1. Introduction

Bearing fault diagnosis plays a crucial role in rotary machines. Accurate detection and isolation of early-stage bearing faults will contribute to more safe and more efficient operation of the rotary machines [1]. An analytic model based approach is an important research issue that is widely applied on fault diagnosis of rotating machinery [2]. At present, data-driven methods (including signal processing, statistical analysis and various advanced AI-model-based methods) are widely applied to bearing health monitoring and fault diagnosis as they can make full use of the vibration information in rotary machines [3,4,5,6,7,8,9,10,11,12,13,14,15,16].

However, the many existing methods are supposed to handle the dataset collected from constant working conditions (i.e., with constant speed or working load). In practice, the working conditions of rotary machines are complex and varying, so the vibration signals are often non-stationary. In recent years, some fault diagnosis methods have been proposed under variable working conditions [17,18].

**Figure 1:**
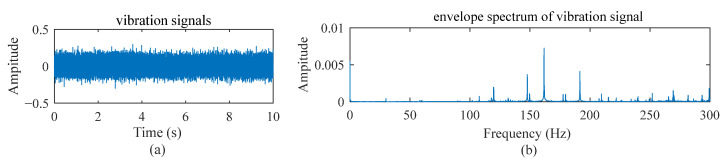
Vibration signal and its envelope spectrum under constant speed working conditions when an inner raceway fault occurs.

**Figure 2:**
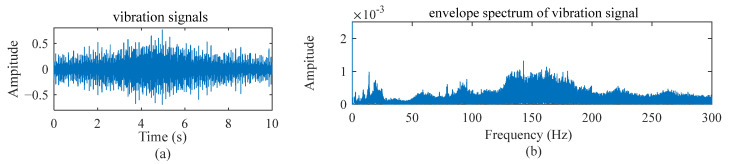
Vibration signal and its envelope spectrum under speed increasing and then decreasing working conditions when an inner raceway fault occurs.

**Figure 3:**
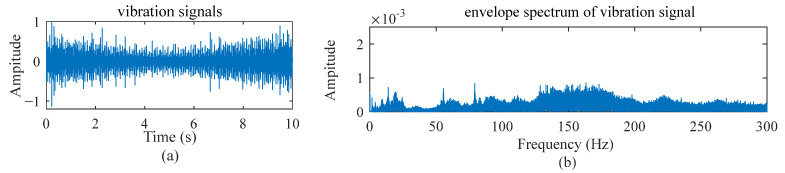
Vibration signal and its envelope spectrum under decreasing speed and then increasing working conditions when an inner raceway fault occurs.

As an example, Figure 1a,b illustrates a vibration signal and its envelope spectrum when an inner raceway fault occurs and the speed setting is a constant value; Figure 2 and Figure 3 show the vibration signals for the cases with two different variable speed settings. It is easy to observe the difference among them. The fault diagnosis model developed for the scenery with constant working conditions is rarely applicable to other sceneries.

Aiming at more practical solutions, many recent studies used signal decomposition techniques [13,14,15], such as VMD [13]. However, the VMD parameters are different under different working conditions. An automatic parameter setting of VMD is essential to solve the issue of variable working conditions. Next to the automatic parameter setting in signal decomposition, it is extremely important to select the right frequency components for fault diagnosis. However, until now, little research has touched on this issue, and there is not enough related research to use data-driven based methods to sort signal components containing important fault information.

This paper proposes a data-driven AVMD-DBN-ELM model for bearing fault diagnosis. First, the WOA-VMD algorithm is used to adaptively decompose vibration signals into different frequency components, and then, an MIV-DBN algorithm is used for the mode sort. After mode selection, reference to the idea of attention mechanisms, and according to the weights obtained by using the MIV algorithm, the diagnosis result of each mode is fused. The DBN-ELM model for fault diagnosis is established by training the selected modes. Finally, a similarity match method is used to match the most similar data, and the parameters are set according to the similar data parameter setting. After acquiring the retained modes, the fault diagnosis model handles these modes and acquires the result of fault diagnosis.

The advantages of the proposed method are as follows. First, the proposed method can decompose vibration signals into different modes adaptively and retain effective modes. The second advantage is that it can learn from the idea of attention mechanism and fuse the results according to the weight of MIV; the third advantage is that it can improve the timeliness of the fault diagnosis.

The rest of this paper is organized as follows. Section 2 mainly describes the process of bearing fault diagnosis. The fault diagnosis experiment is described in Section 3. Section 4 gives the conclusions.

## 2. Methodology

### 2.1. Framework

**Figure 4:**
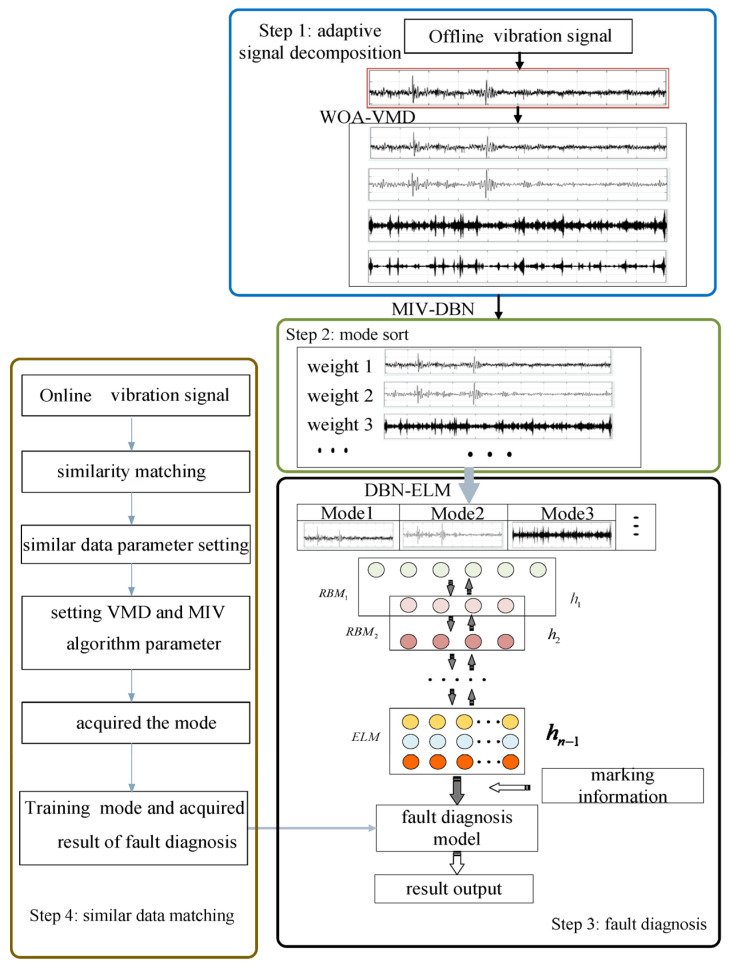
The structure of adaptive VMD and mode sort based DBN-ELM algorithm.

The overview of the proposed adaptive bearing fault diagnosis method is shown in Figure 4. The first step is decomposing vibration signals adaptively. The second step is sorting mode. The third step is training a fault diagnosis model. The fourth step is using a similarity match method to match the most similar data and the fault diagnosis model to acquire the result of the fault diagnosis. This section will describe the detail of the proposed method.

### 2.2. WOA-VMD Method for Decomposing Vibration Signals

VMD is an adaptive and completely non-recursive method and uses signals to decompose modes [13]. The core idea of this method is to build and solve the variational problem. The advantage of this method is that it can effectively decompose vibration signals into different frequency components. Whether the vibration signal decomposition process is completed depends on two main input parameters of VMD: α and *K*, but the value of *α* and *K* may be different under different working conditions. In this paper, the WOA algorithm is used to optimize the values of the two main input parameters.

WOA [19] is an optimization algorithm, and it mimics the bubble-net feeding behavior of humpback whales. It is assumed that the dimension of the data is D and the population has M particles. The coordinate of the i-th particle in space can be described as follows:

(1) $$X_{i}=r\cdot \left(ub-lb\right)+lb$$

where r∈[0,1] is a random number, $X_{i}$ ranges from [lb−ub], lb is the minimum value of the parameter, and ub is the maximum value of the parameter.

When p < 0.5 and |A| < 1, the optimization is performed according to the following equations:

(2) $$\left\{ \begin{array}{l} \vec{D}=\vec{C}\cdot \vec{X^{*}}\left(t\right)-\vec{X}\left(t\right)\\ \vec{X}\left(t+1\right)=\vec{X^{*}}\left(t\right)-\vec{A}\cdot \vec{D}\\ \vec{A}=2\vec{a}\cdot \vec{r_{1}}-\vec{a}\\ \vec{C}=2\cdot \vec{r_{2}}\\ \vec{a}=2-2\left(t/t_{\max}\right) \end{array}\right.$$

where $\vec{r_{1}}$, $\vec{r_{2}}$ and p are random values belonging to [0, 1]; t is the number of the update iteration; $t_{\max}$ is the maximum number of update iteration; $\vec{a}$ is the convergence factor that linearly drops to 0 in the process of iteration; $\vec{X}\left(t\right)$ is the position vector of the current solution; $\vec{X^{*}}\left(t\right)$ is the position vector of the optimal solution; and $\vec{A}$ and $\vec{C}$ are the coefficient vectors.

When p<0.5 and |A|≥1, the optimization is performed according to the following equations:

(3) $$\left\{ \begin{array}{l} \vec{X}\left(t+1\right)={\vec{X}}_{rand}-\vec{A}\cdot \vec{D}\\ \vec{D}=\left|\vec{C}\cdot {\vec{X}}_{rand}-\vec{X}\left(t\right)\right|\\ \vec{A}=2\vec{a}\cdot \vec{r_{1}}-\vec{a}\\ \vec{C}=2\cdot \vec{r_{2}}\\ \vec{a}=2-2\left(t/t_{\max}\right) \end{array}\right.$$

where ${\vec{X}}_{{}_{rand}}$ is a random position vector.

When p≥0.5, the optimization is performed according to the following equations:

(4) $$\left\{ \begin{array}{l} \vec{X}\left(t+1\right)=\vec{D^{\prime }}\cdot e^{bl}\cdot \cos\left(2\pi l\right)+\vec{X^{*}}\left(t\right)\\ \vec{D^{\prime }}=\left|\vec{X^{*}}\left(t\right)-\vec{X}\left(t\right)\right| \end{array}\right.$$

where $\vec{D^{\prime }}$ is the simulation distance from the target, b is the helix constant, and l is a random value belonging to (−1, 1).

Next, we judge whether $t=t_{\max}$ and $\vec{a}$ linearly drop to 0 in the process of iteration. If the termination conditions are not met, we continue the process of optimization. If the termination conditions are met, we output the result of optimization.

The envelope entropy can reflect the sparse nature of the vibration signal. The maximum value of envelope entropy reflects the most uncertain probability distribution (equal probability distribution). The minimum values of envelope entropy are the optimal solution of *α* and *K*. Given the vibration signal x(i)(i=1,2,⋯,N), the envelope entropy $E_{p}$ [13] is:

(5) $$\left\{ \begin{array}{l} E_{p}=-\sum_{i=1}^{N}\varepsilon \left(i\right)\mathrm{lg}\varepsilon \left(i\right)\\ \varepsilon \left(i\right)=\frac{a\left(i\right)}{\sum_{i=1}^{N}a\left(i\right)} \end{array}\right.$$

where a(i) is the envelope signal of the decomposing vibration signal after Hilbert mediation, *N* is the number of sampling points, and ε(i) is a probability distribution sequence through normalized a(i).

The implementation steps of the WOA-VMD algorithm for decomposing vibration signal are as follows: 

Step 1: Pre-process the vibration signals;

Step 2: Set the parameters of WOA;

Step 3: Set the parameters of VMD;

Step 4: Decompose the vibration signal into different frequency components using the VMD algorithm;

Step 5: Judge whether the value of envelope entropy is minimum;

Step 6: According to the judgment result of Step 5, return to Step 3 if the result dissatisfies Step 5. If the result satisfies Step 5, output the result of the variational mode;

Step 7: Complete the process of decomposing the vibration signal adaptively.

### 2.3. Mode Sort Based MIV-DBN Algorithm

The MIV algorithm [20] reflects the change in the weight matrix in the neural network. The MIV algorithm can evaluate the variable correlation. The MIV is an index used to ensure the influence level of input neuron to output neuron. Its symbol represents the direction of correlation. Its absolute value represents the influence level. The detailed calculation process of MIV is described as follows. After the termination of the neural network training, each independent variable value of the training dataset (here named P) increases by 10% to form a new training dataset P1, while each independent variable value of the training data (here named P) reduces by 10% to form a new training dataset P2. P1 and P2 are used as the simulation data of the neural network, and the simulation results A1 and A2 are obtained after neural network training. The difference between A1 and A2 is calculated, where this difference represents the impact value (IV) of output after changing the independent variable value. Then, according to the number of input data, the mean value of VI (MIV) is calculated. According to the above steps, the mean impact value of each independent variable can be calculated. Finally, according to the absolute value of MIV, the mode is sorted to judge the influence level of input data to output neuron, and the process of variable selection is completed.

The second step in Figure 4 shows the process of mode sort based on the MIV-DBN algorithm. The detailed calculation process of the MIV-DBN method is described as follows: 

Step 1: Take the variational mode obtained from the WOA-VMD algorithm as the training dataset;

Step 2: Use the DBN algorithm to train a normal fault diagnosis model;

Step 3: Increase the value of the training dataset (here named P) by 10% to form a new training dataset P1, and reduce the value of the training dataset by 10% to form a new training dataset P2;

Step 4: Use the VMD algorithm to decompose the vibration signal into different frequency components;

Step 5: Based on the P1 and P2, use the fault diagnosis model to obtain the simulation results A1 and A2;

Step 6: Calculate the MIV, and sort the mode according to the absolute value of the MIV to complete the process of mode sorting.

The mode sort can highlight the role of the components that contain fault information and to screen out the parts of the signal that are not useful for diagnosis.

### 2.4. The Process of the DBN-ELM Method to Build the Fault Diagnosis Model

This paper combines the DBN algorithm with the ELM algorithm. The DBN algorithm is used to extract features, while the ELM algorithm is used for classification. The BP algorithm used in the DBN algorithm must be iterative, whereas the use of the ELM algorithm can rapidly learn without iteration. Compared with only using the DBN algorithm, the DBN-ELM method has faster learning speed and better generalization performance, and its structure is shown in step 3 of Figure 4.

The detailed process of the DBN-ELM method is described as follows:

Step 1: Take the selected mode obtained from the MIV-DBN algorithm as the training dataset, and the number of training dataset is equal to the number of selected mode;

Step 2: With different training datasets, the DBN-ELM algorithm is used to train the fault diagnosis model;

Step 3: Refer to the idea of attention mechanisms [21], and according to the weight of MIV, fuse the result in Step 2; 

Step 4: Complete the process to build the fault diagnosis model.

### 2.5. The Process of Online Fault Diagnosis

The above steps solve the problem of establishing a diagnostic model based on offline data, but in fact, the rotating machinery’s continuous operation and online data are generated all the time. Because the online data is generated all the time, the online data fault diagnosis needs to be completed in a timely manner, but the process of parameter optimization takes too much time. Therefore, in this section, a strategy of online diagnosis is simulated, a similarity match method is used to match the most similar data, and the parameters are set according to the similar data parameter setting. This method reduces the time by removing the process of parameter optimization; after acquiring the retained modes, the fault diagnosis model handles these modes and acquires the result of fault diagnosis, and its structure is shown in step 3 of Figure 4. The well-trained fault diagnosis model can diagnose the health conditions of the bearing based on the collected vibration data. The detailed process of online fault diagnosis is described as follows:

Step 1: Use the similarity matching method to match the high similar data, and use the maximum mean discrepancy (MMD) [22] to calculate the distance between online vibration data and offline data;

Step 2: Set the VMD and MIV algorithm parameters according to the most similar data parameter setting;

Step 3: Acquire the modes by using the MIV algorithm;

Step 4: Use the fault diagnosis model to train these modes and acquire the result of fault diagnosis.

This paper proposed an adaptive VMD and mode sort based DBN-ELM algorithm for bearing fault diagnosis. The adaptive decomposition process of vibration signal is completed by using the WOA-VMD method. The MIV algorithm is used to complete the mode sorting process. The DBN-ELM algorithm is used to achieve the establishment of fault diagnosis model. The similarity matching method is used to complete the online fault diagnosis process. Compared with traditional bearing fault diagnosis methods under constant speed working conditions, the proposed method can be applied to more working conditions, and the timeliness of fault diagnosis is also considered.

## 3. Experimental Study

In this section, the bearing fault diagnosis dataset from the University of Ottawa [23] is used to evaluate the proposed method. This section mainly describes the dataset and presents the experimental results. At the same time, several different methods are used for comparative analysis. The average accuracy, standard deviation and time are the list as the evaluation index. Finally, the results are discussed in detail.

### 3.1. Dataset Description and Experimental Setting

In this section, the experimental study is conducted by using the bearing fault diagnosis dataset from the University of Ottawa, in which 5 kinds of faults are considered, and each kind of fault has 4 kinds of time-varying rotational speed working conditions. The 5 kinds of faults includes healthy status (here marked as H), the outer raceway fault (here marked as OF), the inner raceway fault (here marketed as IF), the ball fault (here marked as BF) and a combination fault (here marked as CF). The 4 kinds of working conditions include the speed increasing working conditions (here marked as SI), speed decreasing working conditions (here marked as SD), speed increasing and then decreasing working conditions (here marked as SID), speed decreasing and then increasing working conditions (here marked as SDI). Table 1 describes the experimental dataset.

### 3.2. Experimental Results

#### 3.2.1. Results of Parameter Optimization

The main parameters of VMD are *α* and *K* [14], and whether the vibration signal decomposition process is completed depends on the two main input parameters. Therefore, in this paper, the WOA algorithm is used to optimize the values of the two main input parameters. Different vibration signals using the VMD may have different results. Table 2 lists the results of WOA-VMD. In Table 2, the results of VMD are *α* and *K* are different under different working conditions.

#### 3.2.2. Results of Mode Sort

**Figure 5:**
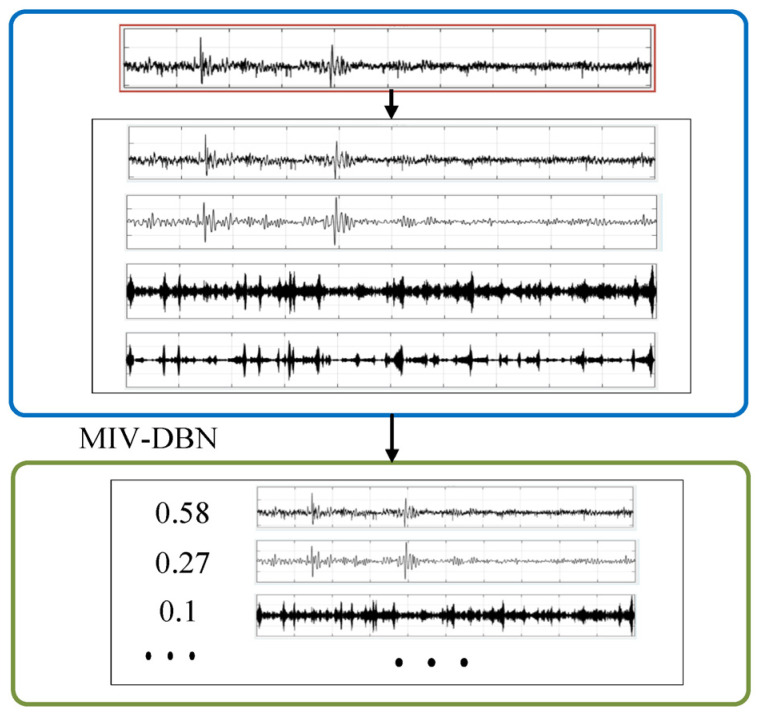
Result of mode sort under the inner raceway fault vibration signal.

After the VMD completes the vibration signal decomposition, the VMD decomposed spectrum is used as the training dataset. The MIV-DBN algorithm is used to train the frequency spectrum data. The MIV value is obtained, and the mode sorting process is completed. Figure 5 shows the results of mode sort, where the absolute values of MIV are the sort basis. Thus, the weights are calculated according to the results of mode sort.

#### 3.2.3. Results of Fault Diagnosis

**Figure 6:**
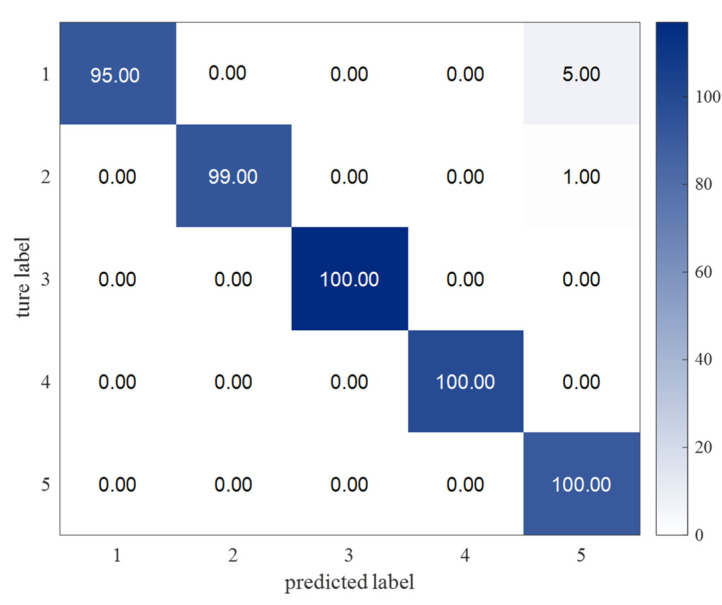
Confusion matrix of fault diagnosis.

**Figure 7:**
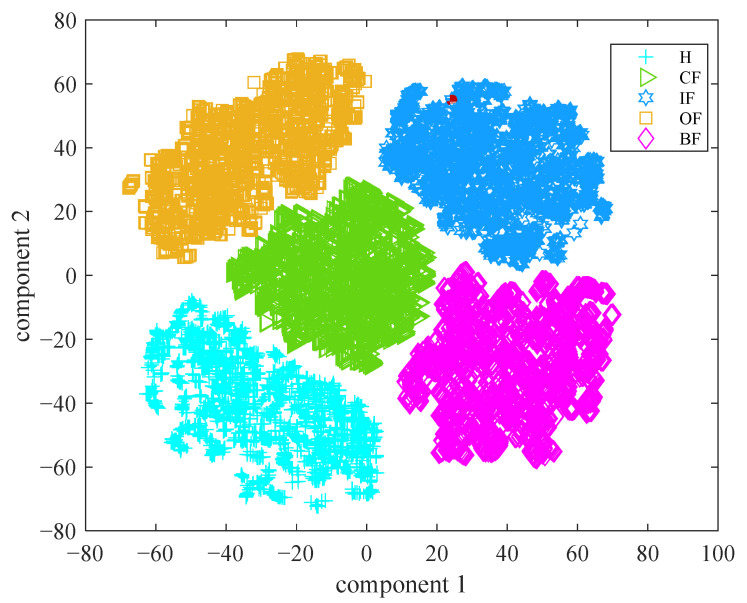
Fault diagnosis accuracy.

**Figure 8:**
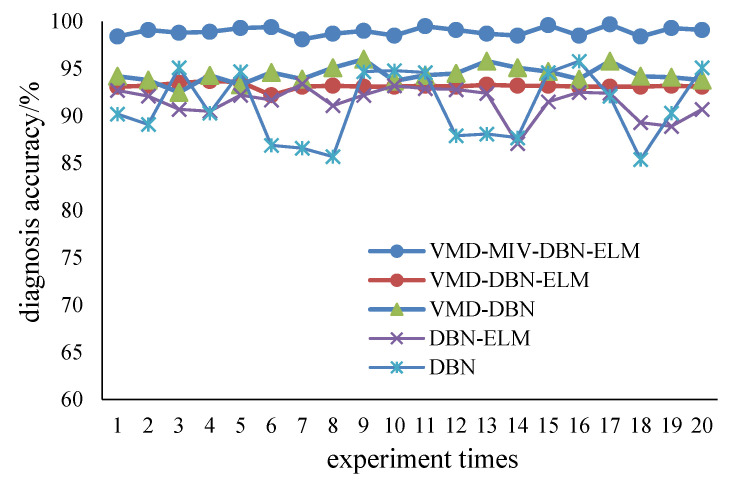
Online fault diagnosis.

With the DBN-ELM, the fault diagnosis accuracies of five kinds of faults are shown in Figure 6. Figure 7 shows the fault diagnosis results of 20 runs. The average diagnosis accuracy is 98.93%, and the standard deviation is 0.288. Different methods (DBN-BP, VMD-DBN-BP, VMD-DBN-ELM and VMD-MIV-DBN-ELM) are used to compare with the method of this paper (DBN-ELM). Table 3 shows the fault diagnosis results. The average accuracy is generally used to evaluate the global accuracy of the model. The standard deviation can be used to measure the stability of model diagnosis. The time in Table 3 can be used to measure the training time of the fault diagnosis model. In this table, the average diagnosis accuracy of the proposed method is higher than those of other methods, and the standard deviation of the proposed method is less than those of other methods. The ELM method reduces the diagnosis time, and the MIV method selects the effective mode, which may be the main reason for the improvement in diagnosis performance. In Figure 8, the online data are marked as red. The MMD is calculated to obtain the similar data parameter setting, finally completing the fault diagnosis; the data belong to inner raceway fault data, and the average accuracy of the fault diagnosis is above 98%. 

## 4. Conclusions

This paper proposes an adaptive VMD and mode sort based DBN-ELM method for bearing fault diagnosis. This method can handle the vibration signals collected from different working conditions in which multiple frequency components are mixed. This method contains four main processes: first, the WOA-VMD is used to decompose the vibration signal into different modes; second, according to the influence level of input neuron to output neuron, the MIV method is used to sort the different modes; third, the DBN-ELM method is used to build the fault diagnosis model; finally, according to the similarity matching method, the process of online fault diagnosis is completed.

The proposed method focuses on the vibration signal collected from complex working conditions in which multiple frequency components are mixed. The method of this paper has three advantages: the first advantage is that it can decompose vibration signal into different modes, and according to the influence level of input neuron to output neuron, the different modes allow sorting. The mode sort can highlight the role of the components that contain fault information and to screen out the parts of the signal that are not useful for diagnosis. The second advantage is that it can learn from the idea of the attention mechanism and fuse the results according to the weight of MIV; the third advantage is that it can improve the timeliness of the fault diagnosis. Therefore, this method is hopeful to be applied in practical industrial conditions to optimize the reliability, accuracy and timeliness of the fault diagnosis.

## Figures and Tables

**Table 1:** Introduction of bearing dataset.

Bearing Health Conditions	Varying Speed Setups	Label	Training Dataset	Testing Dataset
H	SI	1	10,500	4500
	SD		10,500	4500
	SID		10,500	4500
	SDI		10,500	4500
OF	SI	2	10,500	4500
	SD		10,500	4500
	SID		10,500	4500
	SDI		10,500	4500
IF	SI	3	10,500	4500
	SD		10,500	4500
	SID		10,500	4500
	SDI		10,500	4500
BF	SI	4	10,500	4500
	SD		10,500	4500
	SID		10,500	4500
	SDI		10,500	4500
CF	SI	5	10,500	4500
	SD		10,500	4500
	SID		10,500	4500
	SDI		10,500	4500

**Table 2:** Results of WOA-VMD.

Bearing Fault	Number of *K*	Value of *α*
H-SI	7	1997
H-SD	7	1989
H-SID	7	1948
H-SDI	7	2000
OF-SI	7	1995
OF-SD	7	1982
OF-SID	7	1993
OF-SDI	7	1999
IF-SI	7	1956
IF-SD	6	1935
IF-SID	6	1936
IF-SDI	7	1987
BF-SI	7	1980
BF-SD	6	1995
BF-SID	7	1493
BF-SDI	6	1981
CF-SI	7	1946
CF-SD	7	1955
CF-SID	6	1155
CF-SDI	6	1829

**Table 3:** Results of fault diagnosis.

**Method**	**Average Accuracy (%)**	**Standard Deviation**	**Time (s)**
VMD-MIV-DBN-ELM	98.93	0.288	437.65
VMD-DBN-ELM	93.17	0.452	364.37
VMD-DBN	94.38	0.868	583.24
DBN-ELM	91.52	1.605	294.25
DBN-BP	90.99	3.666	388.58

## Data Availability

Not applicable.
